# An inventory of mucin genes in the chicken genome shows that the mucin domain of Muc13 is encoded by multiple exons and that ovomucin is part of a locus of related gel-forming mucins

**DOI:** 10.1186/1471-2164-7-197

**Published:** 2006-08-03

**Authors:** Tiange Lang, Gunnar C Hansson, Tore Samuelsson

**Affiliations:** 1 Department of Medical Biochemistry, Goteborg University, Goteborg, Sweden

## Abstract

**Background:**

Mucins are large glycoproteins that cover epithelial surfaces of the body. All mucins contain at least one PTS domain, a region rich in proline, threonine and serine. Mucins are also characterized by von Willebrand D (VWD) domains or SEA domains. We have developed computational methods to identify mucin genes and proteins based on these properties of the proteins. Using such methods we are able to characterize different organisms where genome sequence is available with respect to their mucin repertoire.

**Results:**

We have here made a comprehensive analysis of potential mucins encoded by the chicken (*Gallus gallus*) genome. Three transmembrane mucins (Muc4, Muc13, and Muc16) and four gel-forming mucins (Muc6, Muc2, Muc5ac, and Muc5b) were identified. The gel-forming mucins are encoded within a locus similar to the corresponding human mucins. However, the chicken has an additional gene inserted between *Muc2 *and *Muc5ac *that encodes the the α-subunit of ovomucin, a protein similar to Muc2, but it is lacking a PTS domain. We also show that the β-subunit of ovomucin is the orthologue of human MUC6. The transmembrane *Muc13 *gene is in chicken as well as in mammals adjacent to the *HEG *(heart of glass) gene. HEG has PTS, EGF and transmembrane domains like Muc13, suggesting that these two proteins are evolutionary related. Unlike previously known mucins, the PTS domain of Muc13 is encoded by multiple exons, where each exon encodes a repeat unit of the PTS domain.

**Conclusion:**

We report new mucin homologues in chicken and this information will aid in understanding the evolution of mucins in vertebrates. The fact that ovomucin, a protein not found in mammals, was located in the same locus as other gel-forming mucins provides strong support that these proteins are evolutionary related. Furthermore, a relationship of HEG and the transmembrane Muc13 is suggested on the basis of their biochemical properties and their presence in the same locus. Finally, our finding that the chicken Muc13 is distributed between multiple exons raises the interesting possibility that the length of the PTS domain could be controlled by alternative splicing.

## Background

The mucosal surfaces are all covered by mucus largely made up of the large glycoproteins referred to as mucins. Mucins play an important role in protection, but some mucins also take part in cell surface signaling and are important for cancer development and progression. Typical for the mucins are the large mucin (PTS) domains rich in the amino acids Ser, Thr and Pro, often characterized by perfect or imperfect tandem repeats [1]. Most mucins also have other characteristic domains such as von Willebrand D (VWD) or SEA (sea urchin sperm protein-enterokinase-agrin) domains. We have developed bioinformatics methods to identify and characterize mucin genes based on these distinct properties of mucins [2]. Using such methods, we recently carried out an analysis of the puffer fish *Fugu rubripes *[2].

There are two major types of mucins, membrane-bound and secreted. In human, nine membrane-bound (MUC1, MUC3A, MUC3B, MUC4, MUC12, MUC13, MUC16 and MUC17) [3-9] and seven secreted mucins (MUC2, MUC5B, MUC5AC, MUC6, MUC7, MUC19 and MUC20) [10-16] have been identified. The secreted mucins can be further sub-divided as being either gel-forming (MUC2, MUC5B, MUC5AC, MUC6 and MUC19) or not (MUC7 and MUC20). The ability to form gels is dependent on the capacity of monomers to form polymeric structures. Gel-forming mucins have three VWD domains in their N-terminal ends that are involved in polymerization through intermolecular disulfide-bonds. They also have a cysteine-knot (CK) domain at their C-terminal ends (reviewed in [17]). The VWD domain was first identified in the prepro-von Willebrand factor [18], hence its name. The gel forming mucins and the von Willebrand factor dimerize with the help of their C-terminal VWD domains in the endoplasmic reticulum (ER) [17-19] and oligomerize through their N-terminal VWD domains in the acidic compartments of the Golgi complex [17,20]. The human transmembrane mucins are all characterized by either a SEA domain or a special variant of the VWD domain that is lacking cysteines. Several of the human transmembrane mucins are known to or predicted to be cleaved in their SEA or VWD domains [21].

To understand the evolution of mucins, we are systematically examining the distribution and structure of mucins in different organisms. The results of such analysis will ultimately provide a better understanding of the function of the human mucins. It is also important to study mucins from organisms such as *C. elegans*, *Drosophila*, zebrafish and mouse as these are important experimental model systems. The previously analyzed puffer fish *Fugu rubripes *[2] has a gene repertoire similar in size to that of man, but according to our analysis it seems to lack several of the mucins found in the human genome. In particular, this is the case for the transmembrane mucins as only one such gene was identified in the fish whereas the human genome encodes at least nine different.

Sequencing and annotating mucin genes is notoriously difficult due to their large size and repetitive nature. Therefore, the identification and classification of putative novel mucins requires a variety of bioinformatics tools as well as expert biological knowledge. Continuing our analysis of animal mucin genes, we now report on novel mucins identified in the chicken genome. Previously, a chicken MUC4-related protein was known and Muc16 was identified by Duraisamy et al [22]. In addition, two chicken mucin-related proteins have been reported and are referred to as the α- and β-subunits of ovomucin, the major component of egg white and responsible for its gel-like properties [23]. We now show that the previously reported β-subunit is the chicken orthologue of human MUC6. We also report on a chicken Muc13 gene. This gene has an unusual organization as the tandem repeats of the PTS domain is encoded by multiple exons where each exon encodes one repeat.

## Results and discussion

### Mucin genes may be reliably predicted using bioinformatics methods

To identify mucins we have used a method (PTSpred) to predict PTS/mucin domains where we analyzed both predicted proteins as well as genomic sequences translated in all six possible reading frames. We have previously applied this method to analyze the puffer fish *Fugu rubripes *[2]. We have here used that method to analyze human and chicken proteins as well as genomic sequences. We have also taken advantage of the fact that all mucins (MUC7 and MUC20 excluded) contain either von Willebrand D (VWD) or SEA domains. Thus, we have analyzed proteins with Pfam models of the VWD and SEA domains and Genewise [24] was used to screen genomic sequences using the same models.

We considered a protein sequence to be a potential mucin if it contained at least one PTS domain as well as a VWD or SEA domain. Such candidates were further evaluated by phylogenetic analyses of SEA/VWD domains. A protein was considered a strong mucin candidate only in case the phylogenetic analysis supported a relationship between its SEA or VWD domain(s) to those of previously characterized mammalian mucins.

To test the efficiency of our computational methods we first analyzed available human protein sequences as well as the human genome assembly (for details see Materials and Methods). All human mucins, except MUC7 and MUC20, contain PTS domains as well as either VWD or SEA domains. In summary, our methods successfully identified all of these previously known mucins. In addition, we identified MUC19 [15,25] that was not known at the time we carried out this work. With the help of EST, mRNA, protein and genome sequences we were also able to reconstruct more complete and accurate human mucin protein sequences and elucidate gene structures (T. Lang et al., unpublished). These results show that our computational methods are reliable in terms of mucin gene predictions and that rigorous analysis of available sequence information is necessary in order to derive reliable predictions of gene and protein sequences.

### Prediction of chicken mucin genes

We have now analyzed the chicken genome for mucin genes making use of the assembled genomic sequence and the proteins predicted by ENSEMBL [26]. The genome assembly used in this work is expected to be approximately 90% complete. We have used methods described above for screening of the human and *F. rubripes *genomes. Most of the VWD and SEA domains identified in searches with hmmer and Genewise could be attributed either to mucins or to other previously known human proteins containing these domains. The predicted chicken mucin genes were characterized by a variety of bioinformatics tools and comparisons with known mucin genes and proteins from other species. For instance, all the sequences of the known mucin genes were aligned to the chicken genome. In this way, we could not only identify the human homologues, but also obtain a more complete sequence and understanding of the predicted chicken mucin. For more information on our current assembly of chicken mucins genes, including a comparison to the ENSEMBL predictions, the reader is referred to our mucin web site [27]. A summary of our current inventory of mucins in man, mouse, chicken and the fish *Fugu rubripes *is shown in Table 1.

**Figure 1:**
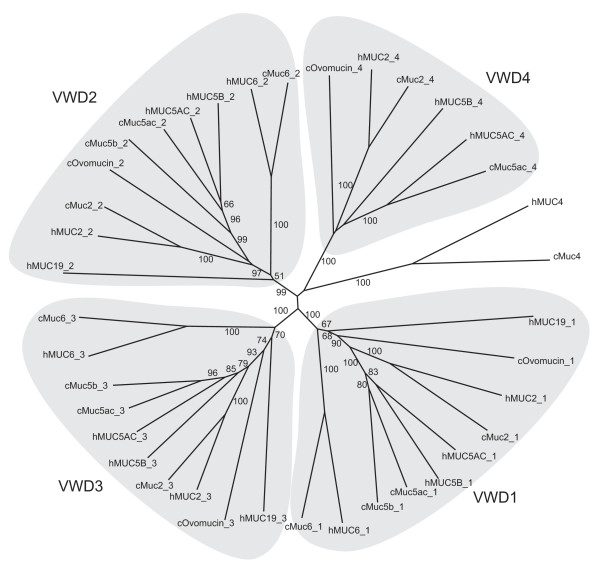
Phylogenetic tree of von Willebrand D domains in human and chicken mucins. A neighbor-joining tree was obtained by ClustalW using 1000 bootstrap replicates. Bootstrap percentages above 50 are shown. Groups containing the VWD1, VWD2, VWD3 and VWD4 domains of mucin type are shown with a shaded background. Animals represented are human (h), mouse (m) and chicken (c).

A total of eight strong mucin candidates in chicken were identified, all with PTS domains and with either VWD (5) or SEA (3) domains. Analysis of the proteins with VWD domains revealed *Muc2*, *Muc5ac*, *Muc5b*, and *Muc6 *genes that are located in a cluster on chicken chromosome 5 and are discussed in more detail below. In addition we found a homologue to Muc4 on chromosome 9. We observed that a protein predicted by ENSEMBL contained the major portion of Muc4, including a part of the PTS domain followed by AMOP, VWD, EGF1, EGF2, and TM domains characteristic for the human MUC4 [6]. The missing N-terminal part, including a signal sequence and the major part of the PTS domain, was reconstructed from the genome sequence. The resulting protein sequence is partially identical to a protein previously described as Muc4-related (Genbank: XP_426704.1) [22]. The VWD domain of the human MUC4 is unusual in that it lacks cysteines, and this is also true for the chicken Muc4. The sequences of the human and chicken VWD domains are similar (Fig. 1) and taken together our information about the chicken protein strongly supports an orthologous relationship to human MUC4.

**Figure 2:**
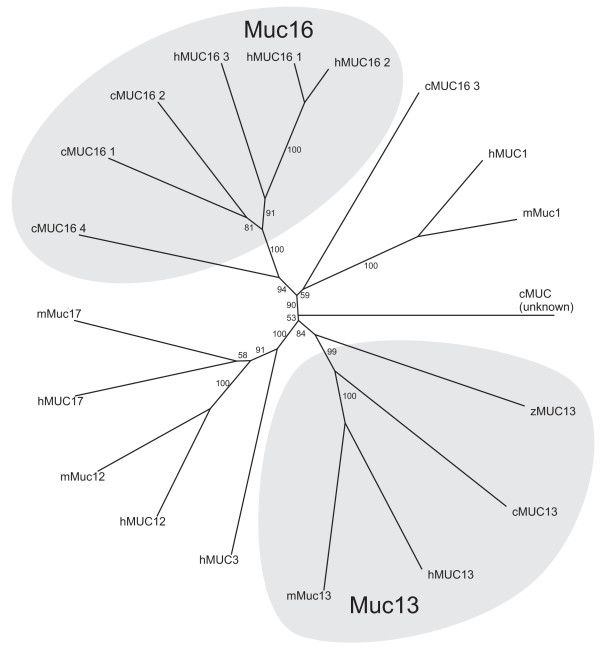
Phylogenetic tree of SEA domains in human, mouse, chicken and zebrafish. A neighbor-joining tree was obtained by ClustalW using 1000 bootstrap replicates. Bootstrap percentages above 50 are shown. The groups containing the Muc1, Muc13 and Muc16 mucins are shown with a shaded background. Animals represented are human (h), mouse (m), chicken (c) and zebrafish (z).

An analysis of predicted proteins with SEA domains identified chicken Muc13 and Muc16 homologues as well as a weak Muc1 candidate. Muc13 is described further below. The chicken Muc16 protein, previously identified by Duraisamy et al [22], is encoded on chromosome 28 and has a PTS domain followed by at least four SEA domains. The assignment as Muc16 based on phylogenetic analysis (Fig. 2) agrees with previous results [22] and is also consistent with the fact that human MUC16 is the only mucin known to have multiple SEA domains [8].

Two different proteins with SEA domains related to human MUC1 were identified. One of these were previously analyzed and it was proposed that it is more closely related to a heparin sulfate proteoglycan than to mammalian Muc1 [22]. The other MUC1-related protein was analyzed here. However, it did not convincingly cluster with the SEA domains of other Muc1 proteins ('unknown' in Fig. 2). Furthermore, the SEA domain of this protein is preceded by a PTS domain, but a transmembrane domain characteristic of MUC1 could not be identified. Finally, the N-terminal region of this putative mucin gene cannot be analyzed due to a gap in the genomic sequence. Therefore, it is not possible at this stage to predict the existence of a chicken Muc1.

We expect the predicted genes to be bona fide mucin genes because of the strong similarity to mucins from other species with respect to protein sequence, protein domain structure as well as gene structure. In general, it is difficult to distinguish between bona fide genes and pseudogenes. However, an analysis of available chicken ESTs provides evidence of expression for a majority of mucins genes that we have identified (Table 1). Thus, only in the case of Muc4, Muc16 and for the protein distantly related to Muc1 we were not able to find a corresponding EST sequence. The absence of EST support is not conclusive, as the available chicken EST data is not expected to be comprehensive.

At the same time it must be pointed out that there are limitations to our approach. We are not able to effectively identify mucins that are lacking VWD and SEA domains, mainly because that PTSpred will give rise to a number of false positive sequences. In addition, we might fail to detect mucin candidates because genome assemblies are incomplete, particularly with respect to mucin genes, and because of limitations in gene prediction procedures.

### The ovomucin gene is part of a gene cluster with gel-forming mucins Muc2, Muc5ac, Muc5b and Muc6

**Figure 3:**
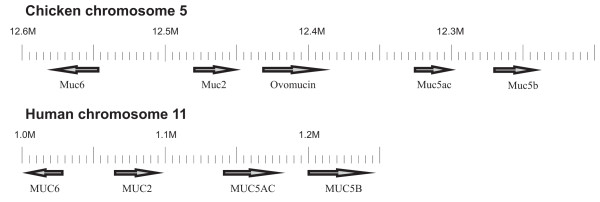
Organization of the gene cluster for gel-forming mucins in chicken as compared to man. The orientation of the genes is indicated by arrows. The major difference between the two organisms is the presence of the ovomucin gene in chicken.

Five VWD-containing proteins were found within a region of chromosome 5, covering 12 million bases. The domain structure of the proteins on chromosome 5 suggested that this region has an organization similar to the human *MUC2/5AC/5B/6 *gene cluster. The relative gene order and polarity was identical to the corresponding human mucins as shown in Fig. 3. Thus, the *Muc6 *mucin is positioned next to and in the opposite direction to *Muc2*, *Muc5ac *and *Muc5b*.

**Figure 4:**
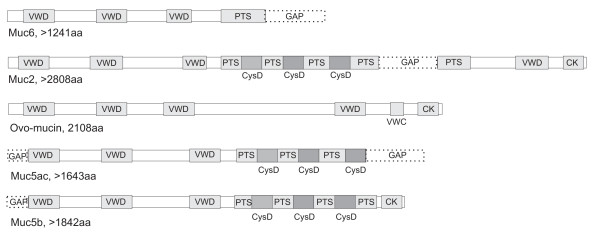
Domain organization of mucins in the chicken gel-forming mucin cluster. Dotted lines indicate a gap in the genome assembly and when such gaps occur, a minimum size of the protein is indicated.

The domain structures of the individual chicken gel-forming mucins were analyzed and the results are shown in Fig. 4. Typically, these mucins have three VWD domains followed by alternating PTS and CysD domains, and at the C-terminal end a cysteine-knot (CK) domain. The Muc2 and ovomucin proteins have an additional VWD domain. The chicken Muc2 ortholog was identified as the protein most similar to the human MUC2. However, the central part of the predicted molecule contained at least three CysD and four PTS domains, whereas the human protein only has two CysD and two PTS domains. A gap in the genomic sequence precludes further comparison and a conclusion as to the differences. The chicken Muc5ac and Muc5b proteins have a similar domain structure with central repeated CysD and PTS domains as in the human orthologues. However, chicken Muc5B lacks the C-terminal VWD domain in contrast to the human orthologue. The chicken Muc5ac genomic sequence has a large gap in its 3'-end preventing further comparison. Also for Muc6 the domain structure is identical to the human orthologue (Fig. 4), but a gap in the 3' genomic sequence makes it impossible to compare this region.

All VWD domains identified by a screen of the chicken genome with Genewise were also compared to previously known VWD domains using BLAST and ClustalW. The phylogenetic tree from a ClustalW analysis is shown in Fig. 1. Interestingly, all the mucin VWD domains are clustered in a characteristic manner based on their position in the mucins as we have previously shown for *Fugu rubripes *mucins [2]. The different VWD domains numbered 1–4 in Fig. 1 are clearly homologous such that the chicken VWD-1 is most closely related to the human VWD-1, etc. The grouping of the human and chicken VWD domains strongly supports our assignment of the chicken mucins as Muc2, Muc5ac, Muc5b and Muc6, respectively.

When the chromosome 5 locus of chicken is compared to the corresponding locus in human, the most notable difference is the presence of one additional gene in the chicken. The predicted protein contains four VWD domains organized as for the gel-forming mucins (Fig. 4). This protein was recently cloned by Watanabe *et al *[23] and referred to as the α-subunit of ovomucin. An additional subunit called β has also been described [23]. Both subunits are abundant in egg white and are responsible for its gel-like properties. However, from the results presented here it is obvious that the β-subunit of ovomucin is an orthologue to the human MUC6. In the following, the α-subunit of ovomucin is simply referred to as ovomucin as this protein is specific to the chicken mucin locus. Ovomucin has a similar domain structure as the other proteins in the cluster except that it does not contain the PTS domain characteristic of mucins (Fig. 4).

Interestingly, from the phylogenetic tree in Fig. 1 it seems that the VWD domains of ovomucin are more deeply branched than Muc2/5ac/5b, suggesting that this ovomucin is a more ancient protein. It will be interesting to further study this issue by identifying homologues of the gel-forming mucins in other species. Preliminary results suggest that there are ovomucin homologues in *X. tropicalis *and in the fishes *F. rubripes*, *T. nigroviridis *and *D. rerio *(zebrafish). However, ovomucin is not present in man and rodents. The tree in Fig. 1 also seems to suggest that Muc6 is more deeply branched than the other gel-forming mucins of the same locus, raising the possibility that this protein is the ancestral form of the Muc2/5ac/5b/Muc6 proteins.

### The PTS domain of Muc13 is encoded by multiple exons where each exon corresponds to a repeated unit

**Figure 5:**
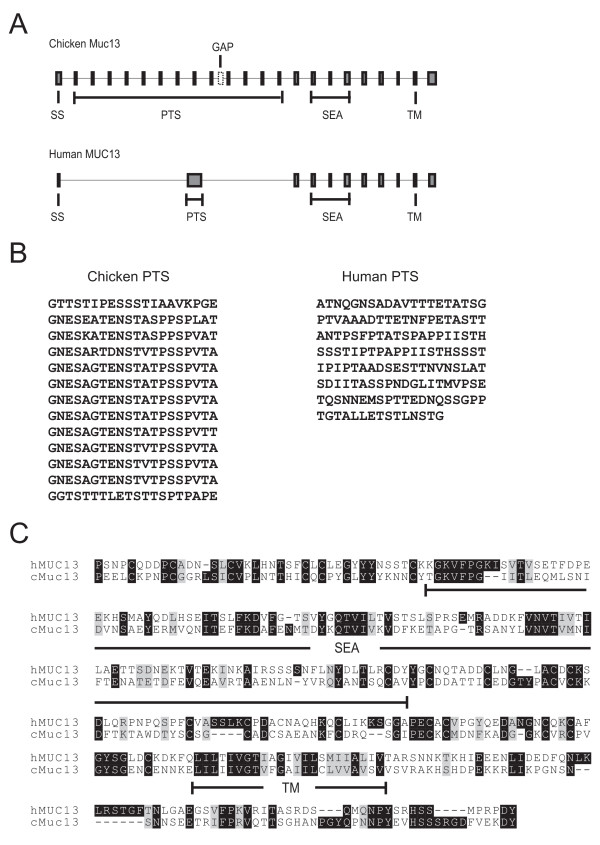
Comparison of the MUC13 sequence in man and chicken. *A*. Genomic organization of exons and introns and the domains encoded by the exons. *B*. Amino acid sequences of the PTS (mucin) domains of the chicken and human MUC13. For the chicken PTS each line corresponds to one exon. *C*. Alignment of the amino acid sequence C-terminal of the PTS domain. Identical and similar amino acids are indicated with black and grey, respectively, and domains are shown under each sequence.

A gene encoding the chicken Muc13 orthologue was identified on chromosome 7. The protein has an N-terminal signal sequence, followed by one PTS, one SEA, one transmembrane domain and a cytoplasmic tail. There is a gap in the genome assembly encoding the PTS domain and therefore the full sequence of this domain cannot be predicted. However, the PTS domain is composed of at least 12 repeats, each 20 amino acids in length (Fig. 5).

Typical for the PTS domains of previously known mucins are that these are built from tandem repeats that often show a remarkable length polymorphism (VNTR, variable number of tandem repeats) [1,28]. The mechanism and functional significance of this variability in length is currently not known, but there are several indications that such variation is associated with disease. The allele length of *MUC1 *has been linked to susceptibility to *Helicobacter pylori *infection and gastric cancer [29,30]. Furthermore, it has recently been suggested that the allele length of *MUC1 *influences the expression of tumor associated carbohydrate antigens and possibly also the aggressiveness of gastric cancer [31].

For all previously described mucins, including the human MUC13, the PTS domain is found within a single large exon. However, the chicken Muc13 PTS domain is encoded by multiple exons. There is a chicken EST (Genbank accession AJ452523) that gives support to this conclusion. As with most other mucins, the chicken Muc13 PTS domain contains repeats. It is interesting to note that the sequences encoded by the exons are nearly identical, i.e. the sequence encoded by one exon corresponds to a repeat unit of the PTS domain (Fig. 5).

The chicken Muc13 tandem repeats thus have a different genomic organization as compared to higher animals. An analysis of zebrafish proteins (unpublished) identified a Muc13 homologue (Fig. 2) with its gene encoding the PTS domain divided into several exons. These findings suggest that this organization of the PTS domain represents an ancestral design of the vertebrate Muc13 gene and perhaps of other mucins.

The genomic organization of the Muc13 gene raises the possibility that a variation in length of the PTS domain may be accomplished not only by recombination events, as is the case for the human MUC1 polymorphism, but also by a regulation of splicing of mucin domain exons. This will allow a length variation not only between individuals, but also within one and the same individual.

### Relationship of Muc13 and HEG

The HEG (heart of glass) gene was first identified in zebrafish where it regulates the growth of the heart [32]. Three isoforms are generated, one of which is predicted to be transmembrane and two secreted. Homologues have been identified in vertebrates, including man, and we now identified a chicken homologue. We observed that this protein is encoded by a gene adjacent to the *Muc13 *gene on chromosome 7 and these two genes have the same polarity. Synteny between chicken and man extends even beyond these two genes in both directions. Analysis of the human and mouse genomes shows that the *HEG *and *Muc13 *genes are organized in the same manner in these animals. Although Muc13 has an SEA domain which is absent in HEG, there are interesting similiarities between the two proteins as both have transmembrane, EGF and PTS domains. This observation together with the fact that the genes are in the same locus suggest an evolutionary relationship.

### The evolution of vertebrate mucins

The results of our inventory of mucins in vertebrates are summarized in Table 1. In our previous study of *F. rubripes *mucins, we concluded that this vertebrate has a set of gel-forming mucins comparable to those of man and rodents. Further analysis of *F. rubripes*, *Tetraodon nigroviridis*, and *Danio rerio *as well as *Ciona intestinalis *suggest that some of the mucins are quite different to the higher vertebrate mucins (in preparation). In the chicken however, we found obvious homologues of the primate and rodent Muc2, Muc5ac, Muc5b, and Muc6. They are homologous both with respect to sequence of the VWD domains as well as to their localization and direction in the gene cluster. Interestingly, the chicken cluster contains an additional gene encoding ovomucin, found in egg white. This gene seems to be present also in frogs and fishes (Lang, T., et al., unpublished observation), but has disappeared during the development of mammals and might not be needed by animals where the fertilized egg is developed within the female body. A more detailed study of the phylogenetic distribution of ovomucin will probably give more clues as to its evolutionary history.

Whereas fish, chicken, and man have a reasonably similar set of gel-forming mucins, many of the transmembrane type mucins are missing in chicken and fish. In particular, this is true for fish as only genes encoding Muc13 and a MUC1-like protein was identified in *F. rubripes*. In the chicken, also the transmembrane mucins Muc4 and Muc16 were identified. Still the transmembrane mucins homologous to the human MUC3, MUC12, and MUC17 seem to be missing in chicken as well as in fishes. These mucins might therefore be a more recent development in vertebrates. A more detailed view of the phylogeny of these proteins will be crucial to better understand the evolution of mucins. Therefore, it is necessary to carry out a careful inventory of mucins in more animals.

## Conclusion

We have identified several novel mucin homologues in chicken. We have shown that chicken has a set of mucins comparable to that of human although we fail to identify a homologue to the gel-forming MUC19 and to the transmembrane MUC3, MUC12, MUC15 and MUC17 proteins.

Ovomucin, similar to Muc2 but without a PTS domain, is a protein found in chicken but not in mammals. We now have shown that the gene encoding ovomucin is part of a locus highly homologous to a human locus containing the *Muc6*, *Muc2*, *Muc5ac*, and *Muc5b *genes. We have also demonstrated that the protein referred to as the β-subunit of ovomucin is a protein homologous to human MUC6.

The chicken transmembrane mucin Muc13, as well as the homologues in man and mouse, contains SEA, EGF and PTS domains on the extracellular side of the membrane. Both in chicken and mammals the *HEG *gene was found to be located adjacent to the *Muc13 *gene. HEG is a transmembrane protein with EGF and PTS domains as Muc13, although no SEA domain can be identified in HEG. Therefore, an evolutionary relationship between Muc13 and HEG is implied.

Finally, we have shown that the PTS domain of Muc13 is encoded by multiple exons, where each exon encodes a repeat unit of the PTS domain. This is in contrast to previously described PTS domains that are all encoded by one exon only. Allelic polymorphism affecting the length of the PTS domain is observed in human mucins. The gene organization in chicken suggests that a variability in the PTS domain could also be accomplished within an individual through alternative splicing.

## Methods

### Sources of sequence information

As sources for protein and genomic sequences we used UCSC [33], Ensembl [26], NCBI [34], and Celera [35]. Protein domain profiles were from Pfam [36]. We made use of the ENSEMBL version of the chicken genome, 27.1d. The genomic DNA sequence had 111864 contigs, with a total of 1,08 × 10^9 ^bases.

### Bioinformatic methods

For identification of PTS domains we made use of PTSpred that can be used to search both DNA and protein sequences [2]. To identify Pfam domains we used for protein sequences hmmpfam of the hmmer package [37] and for nucleotide sequences Genewise [24]. Transmembrane domains were identified by TMHMM [38] and signal sequences by SignalP [39]. For exon prediction Genscan [40] was used. Alignments of proteins and DNA were done by BLAST, ClustalW [41] or programs of the GCG package (GCG, Madison, WI). The repetitive nature of the PTS domains was analyzed with Dotplot of the GCG package. In house Perl scripts were used for additional tasks.

### Analysis of chicken PTS, VWD and SEA domains of chicken

Two different sets of proteins were considered, on the one hand, proteins predicted by ENSEMBL and on the other hand, proteins predicted by *ab initio *methods. When analyzing the ENSEMBL proteins the PTS domain, VWD and SEA domain analysis identified 146, 53 and 26 proteins, respectively. Analysis of the corresponding set of proteins predicted by ab initio methods resulted in 78 PTS, 52 VWD and 17 SEA domain candidates. Ten different proteins had both PTS and either VWD or SEA domains. Two were identified as related to otogelin. The remaining eight proteins were identified as Muc1 (weak candidate), Muc2, Muc4, Muc5ac, Muc5b, Muc6, Muc13, and Muc16 and are described under Results and Discussion. We also used Genewise to scan the chicken genome sequence but that analysis did not result in any additional strong mucin candidates.

## Authors' contributions

TL carried out all bioinformatics analyses and prepared all figures. GH and TS conceived of the study and drafted the manuscript jointly. All authors read and approved the final manuscript.

## Figures and Tables

**Table 1:** Mucins identified in man, mouse, chicken and puffer fish

Mucin	Type^a^	Characteristic Pfam domain	Chicken	Human	Mouse	*F. rubripes*
MUC1	TM	SEA	? (0/10)^d^	+	+	?
MUC2	G	VWD	+ (23/53)	+	+	+^b^
MUC3	TM	SEA		+	+?^c^	
MUC4	TM	VWD	+ (0/23)	+	+	
MUC5AC	G	VWD	+ (8/42)	+	+	
MUC5B	G	VWD	+ (11/37)	+	+	
MUC6	G	VWD	+ (19/31)	+	+	
MUC7				+		
MUC10					+	
MUC12	TM	SEA		+	+?^c^	
MUC13	TM	SEA	+ (13/24)	+	+	+
MUC14					+	
MUC15	TM			+		
MUC16	TM	SEA	+ (0/16)	+	+	
MUC17	TM	SEA		+	+?^c^	
MUC19	G	VWD		+	+	
MUC20				+	+	
Ovomucin		VWD	+ (45/45)			

^a ^'TM' refers to transmembrane domain and 'G' gel-forming mucin

^b^All VWD-containing mucins in *Fugu rubripes *were named Muc2, although the evolutionary relationship of these mucins to the human mucins MUC2/5AC/5B/6 is not clear.

^c^The mucin gene cluster in mouse for the *Muc3/Muc12/Muc17 *mucins is incompletely sequenced. A mouse mucin has been described as Muc3 [42], but is most likely the orthologue of the human MUC17.

^d^The numbers within parentheses indicate the number of exons supported by chicken ESTs as compared to the total number of exons. For Muc1, Muc5ac, and Muc16 the indicated total number of exons is a minimum number as the complete gene structure is not known.

## References

[B1] Hollingsworth MA, Swanson BJ (2004). Mucin in cancer: protection and control of the cell surface. Nat Rev Cancer.

[B2] Lang T, Alexandersson M, Hansson GC, Samuelsson T (2004). Bioinformatic identification of polymerizing and transmembrane mucins in the puffer fish Fugu rubripes.. Glycobiology.

[B3] Gendler SJ, Lancaster CA, Taylor-Papadimitriou J, Duhig T, Peat N, Burchell J, Pemberton L, Lalani EN, Wilson D (1990). Molecular cloning and expression of human tumor-associated polymorphic epithelial mucin. J Biol Chem.

[B4] Pratt WS, Crawley S, Hicks J, Ho J, Nash M, Kim YS, Gum JR, Swallow DM (2000). Multiple transcripts of MUC3: Evidence for two genes MUC3A and MUC3B. Biochem Biophys Res Commun.

[B5] Williams SJ, McGuckin MA, Gotley DC, Eyre HJ, Sutherland GR, Antalis TM (1999). Two novel mucin genes down-regulated in colorectal cancer identified by differential display. Cancer Res.

[B6] Moniaux N, Nollet S, Degand P, Laine A, Aubert JP (1999). Complete sequence of the human mucin MUC4: a putative cell membrane-associated mucin. Biochem J.

[B7] Williams SJ, Wreschner DH, Tran M, Eyre HJ, Sutherland GR, McGuckin MA (2001). MUC13, a novel human cell surface mucin expressed by epithelial and hemopoietic cells. J Biol Chem.

[B8] Yin BWT, Dnistrian A, Lloyd KO (2002). Ovarian cancer antigen CA125 is encoded by the MUC16 mucin gene. Int J Cancer.

[B9] Gum JR, Crawley SC, Hicks JW, Szymkowski DE, Kim YS (2002). MUC17, a novel membrane-tethered mucin. Biochem Biophys Res Commun.

[B10] Gum JR, Hicks JW, Toribara NW, Siddiki B, Kim YS (1994). Molecular cloning of human intestinal mucin (MUC2) cDNA. Identification of the amino terminus and overall sequence similarity to prepro-von Willebrand factor. J Biol Chem.

[B11] Desseyn JL, Buisine MP, Porchet N, Aubert JP, Laine A (1998). Genomic organization of the human mucin gene MUC5B-cDNA and genomic sequences upstream of the large central exon. J Biol Chem.

[B12] Li DZ, Gallup M, Fan N, Szymkowski DE, Basbaum CB (1998). Cloning of the amino-terminal and 5'-flanking region of the human MUC5AC mucin gene and transcriptional up-regulation by bacterial exoproducts. J Biol Chem.

[B13] Toribara NW, Roberton AM, HO SB, Kuo WL, Gum E, Hicks JW, Gum JR, Byrd JC, Siddiki B, Kim YS (1993). Human gastric mucin. J Biol Chem.

[B14] Bobek LA, Tsai H, Biesbrock AR, Levine MJ (1993). Molecular cloning, sequence, and specificity of expression of the gene encoding the low molecular weight human salivary mucin (MUC7). J Biol Chem.

[B15] Chen Y, Zhao YH, Kalaslavadi TB, Hamati E, Nehrke K, Le AD, Ann DK, Wu R (2004). Genome-Wide Search and Identification of a Novel Gel-Forming Mucin MUC19/Muc19 in Glandular Tissues. Am J Resp Cell Mol Biol.

[B16] Higuchi T, Orita T, Katsuya K, Yamasaki Y, Akiyama K, Li H, Yamamoto T, Saito Y, Nakamura M (2004). MUC20 Suppresses the Hepatocyte Growth Factor-Induced Grb2-Ras Pathway by Binding to a Multifunctional Docking Site of Met. Mol Cell Biol.

[B17] Perez-Vilar J, Hill RL (1999). The structure and assembly of secreted mucins. J Biol Chem.

[B18] Sadler JE (1991). von Willebrand factor. J Biol Chem.

[B19] Asker N, Axelsson MAB, Olofsson SO, Hansson GC (1998). Dimerization of the human MUC2 mucin in the endoplasmic reticulum is followed by a N-glycosylation-dependent transfer of the mono- and dimers to the Golgi apparatus. J Biol Chem.

[B20] Axelsson MAB, Asker N, Hansson GC (1998). O-glycosylated MUC2 monomer and dimer from LS 174T cells are water-soluble, whereas larger MUC2 species formed early during biosynthesis are insoluble and contain nonreducible intermolecular bonds. J Biol Chem.

[B21] Macao B, Johansson DGA, Hansson GC, Härd T (2006). Auto-proteolysis coupled to protein folding in the SEA domain of the membrane-bound MUC1 mucin. Nature Struct Mol Biol.

[B22] Duraisamy S, Ramasamy S, Kharbanda S, Kufe D (2006). Distinct evolution of the human carcinoma-associated transmembrane mucins, MUC1, MUC4 AND MUC16. Gene.

[B23] Watanabe K, Shimoyamada M, Onizuka T, Akiyama H, Niwa M, Ido T, Tsuge Y (2004). Amino acid sequence of a-subunit in hen egg white ovomucin deduced from cloned cDNA. DNA Sequence.

[B24] Birney E, Clamp M, Durbin R (2004). GeneWise and Genomewise. Genome Res.

[B25] Culp DJ, Latchney LR, Fallon MA, Denny PA, Denny PC, Couwenhoven RI, Chuang S (2004). The Gene Encoding Mouse Muc19: cDNA, Genomic Organization and Relationship to Smgc. Physiol Genomics.

[B26] Ensembl [http://www.ensembl.org/].

[B27] Mucin web site [http://www.medkem.gu.se/mucinbiology/databases].

[B28] Gendler S, Griffiths B, Corney G, Taylor-Papadimitriou J, Bramwell ME, Swallow.DM (1987). The human tumour-associated epithelial mucins are coded by an expressed hypervariable gene locus PUM. Nature.

[B29] Silva F, Carvalho F, Peixoto A, Seixas M, Almeida R, Carneiro F, Mesquira P, Figueiredo C, Nogeira C, Swallow DM, Amorim A, David L (2001). MUC1 gene polymorphism in the gastric carcinogenesis pathway. Eur J Human Gen.

[B30] Vinall LE, King M, Novelli M, Green CA, Daniels G, Hilkens J, Sarner M, Swallow DM (2002). Altered expression and allelic association of the hypervariable membrane mucin MUC1 in Helicobacter pylori gastritis. Gastroenterology.

[B31] Santos-Silva F, Fonseca A, Caffrey T, Carvalho F, Mesquita P, Reis C, Almeida R, David L, Hollingsworth MA (2005). Thomsen-Friedenreich antigen expression in gastric carcinomas is associated with MUC1 mucin VNTR polymorphism. Glycobiology.

[B32] Mably JD, Mohideen MA, Burns CG, Chen JN, Fishman MC (2003). heart of glass regulates the concentric growth of the heart in zebrafish. Curr Biol.

[B33] UCSC Genome Bioinformatics [http://genome.ucsc.edu/].

[B34] Ensembl [http://www.ensembl.org/].

[B35] NCBI, National Center for Biotechnology information [http://www.ncbi.nlm.nih.gov/].

[B36] Celera Genomics [http://www.celera.com/].

[B37] Pfam [http://www.sanger.ac.uk/Software/Pfam/].

[B38] HMMER: profile HMMs for protein sequence analysis [http://hmmer.wustl.edu/].

[B39] Sonnhammer EL, von Heijne G, Krogh A (1998). A hidden Markov model for predicting transmembrane helices in protein sequences. Proc Int Conf Intell Syst Mol Biol.

[B40] Nielsen H, Engelbrecht J, Brunak S, von Heijne G (1997). Identification of prokaryotic and eukaryotic signal peptides and prediction of their cleavage sites. Prot Eng.

[B41] Burge C, Karlin S (1997). Prediction of complete gene structures in human genomic DNA. J Mol Biol.

[B42] Thompson JD, Higgins DG, Gibson TJ (1994). CLUSTAL W: improving the sensitivity of progressive multiple sequence alignment through sequence weighting, position-specific gap penalities and weight matrix choice. Nucl Acid Res.

[B43] Khatri IA, Forstner GG, Forstner JF (1997). The carboxyl-terminal sequence of rat intestinal mucin RMuc3 contains a putative transmembrane region and two EGF-like motifs. Bioch Biophys Acta.

